# Ileal Bile Acid Transporter Inhibitor Improves Hepatic Steatosis by Ameliorating Gut Microbiota Dysbiosis in NAFLD Model Mice

**DOI:** 10.1128/mBio.01155-21

**Published:** 2021-07-06

**Authors:** Masahiro Matsui, Shinya Fukunishi, Takashi Nakano, Takaaki Ueno, Kazuhide Higuchi, Akira Asai

**Affiliations:** a Second Department of Internal Medicine, Osaka Medical College, Takatsuki, Japan; b Department of Microbiology and Infection Control, Osaka Medical College, Takatsuki, Japan; c Department of Oral Surgery, Osaka Medical College, Takatsuki, Japan; Washington University School of Medicine; Washington University School of Medicine

**Keywords:** nonalcoholic fatty liver disease, ileal bile acid transporter inhibitor, gut microbiome, nonalcoholic steatohepatitis

## Abstract

Nonalcoholic fatty liver disease (NAFLD), characterized by excessive fat deposition in the liver unrelated to alcohol consumption, is highly prevalent worldwide. However, effective therapeutic agents approved for NAFLD treatment are lacking. An ileal bile acid transporter inhibitor (IBATi), which represents a new mode of treatment of chronic idiopathic constipation, leads to increased delivery of bile acids to the colon. We investigated the effect of IBATi against NAFLD through modification of the gut microbiota in mice. IBATi treatment significantly suppressed body weight gain, liver dysfunction, and serum low-density lipoprotein levels and significantly decreased NAFLD activity scores in high-fat diet (HFD) mice. Treatment with IBATi ameliorated the decreased hepatic cholesterol 7-a-monooxygenase (*Cyp7a1*) and increased ileal fibroblast growth factor 15 (*Fgf15*) mRNA expression in HFD mice. Further, IBATi treatment changed the α-diversity in the gut microbiota reduced by HFD, which was analyzed in feces using 16S rRNA sequencing. To establish the mechanism underlying improvement in NAFLD induced by IBATi, we recolonized antibiotic solution-treated mice by fecal microbiome transplantation (FMT) using stool from HFD or HFD plus IBATi mice. This is the first report that fecally transplanted gut microbiota from HFD plus IBATi mice prevented hepatic steatosis caused by HFD. In conclusion, IBATi improved hepatic steatosis by ameliorating gut microbiota dysbiosis in NAFLD model mice, suggesting a potential therapeutic agent for NAFLD treatment.

## INTRODUCTION

Dramatic lifestyle changes in the past century have radically altered health priorities in most areas of the world, owing to the growing incidence of noncommunicable diseases. The current epidemic of chronic liver disease is related to the burden of nonalcoholic fatty liver disease (NAFLD) (1, 2), which is characterized by excessive fat deposition in the liver in the absence of alcohol consumption. The most common risk factors of NAFLD include obesity, insulin resistance, and features of metabolic syndrome. Community surveys, based on ultrasonography or proton nuclear magnetic resonance (NMR) spectroscopy, have assessed the highest prevalence of NAFLD in South America (31%) and the Middle East (32%), followed by Asia (27%), the United States (24%), and Europe (23%), whereas NAFLD is less common in Africa (14%) (1). Most liver-related outcomes occur once cirrhosis has developed, with the exception of hepatocellular carcinoma (HCC) that can develop even in the absence of cirrhosis, and nonalcoholic steatohepatitis, which is rapidly increasing as an etiology of liver cirrhosis (3).

The first line of treatment for NAFLD is lifestyle modification to achieve weight reduction, particularly through diet and exercise. However, weight reduction is difficult to achieve and maintain, and pharmacological agents approved for the treatment of NAFLD are lacking (4). The ileal bile acid transporter (IBAT), also called the apical sodium-dependent bile acid transporter (ASBT), is a key element in the enterohepatic circulation of bile acids (BAs). Treatment with a locally acting IBAT inhibitor (IBATi) led to increased delivery of BAs to the colon, representing a new mode of treatment for chronic idiopathic constipation (5). It was recently reported that inhibition of ileal BA uptake protected against NAFLD in mice (6). However, the effect of IBATi against NAFLD through the gut microbiota is unclear.

The intestine and liver are anatomically linked via the hepatic portal system, also termed the gut-liver axis. The gut microbiota and their metabolic products may influence liver pathology, and this axis plays a pivotal role in the pathogenesis of NAFLD (7, 8). The human gut microbiota contains over 100-fold more genes than its host and has also been suggested to be an important environmental factor involved in the pathogenesis of NAFLD (9). However, how IBATi affects the gut microbiota in NAFLD has yet to be reported. It is known that the gut microbiota varies as well in different populations globally; however, some studies of gut microbiota in NAFLD from different countries have reported similar dysbiosis (10–13). If the administration of IBATi can change this gut microbiota dysbiosis in NAFLD, IBATi may be effective in NAFLD in various populations.

Gut microbiota composition is partially modulated by extracellular metabolites derived from the host and modified by microbes. Among these, BAs constitute a highly abundant pool of host-derived and microbial-modified metabolites that are major regulators of the gut microbiota. BAs are synthesized from cholesterol in the liver, secreted into bile as the major solute, and facilitate lipid absorption in the intestine. Approximately 95% of intestinal BAs are reabsorbed in the ileum by the IBAT and conveyed through the portal vein to the liver, where they are taken up by hepatocytes to be resecreted into the bile. BAs are antibacterial and exert strong selective forces on the intestinal microbiota. Even within a single bacterial species, there can be differential sensitivity to specific BAs. Dietary fat content can regulate the transit time and amount of secreted bile, thus shaping the microbiota (14). In this study, we investigated the effect of IBATi against NAFLD through modification of the mouse gut microbiota in an effort to identify a novel therapeutic agent for treatment of NAFLD.

## RESULTS

### Effects of IBATi on body weight and hepatic steatosis in HFD mice.

Six-week-old mice were fed either the control diet or high-fat diet (HFD) for 12 weeks. During the experiment, the average food intake of mice in the HFD plus IBATi and control diet groups did not differ. As shown in Fig. S1A and B in the supplemental material, body weights were significantly higher in the HFD groups than in the control diet groups after 6 weeks of treatment. This difference was maintained until the end of the study. The average body weight gain of the HFD plus IBATi group was significantly suppressed during the 6- to 12-week period. The average liver weight/body weight ratio was significantly higher in the HFD groups than in the control diet groups, which was inhibited by treatment with IBATi. After 12 weeks, livers were excised from each group and examined by hematoxylin and eosin (H&E) and oil red O staining. Although the liver sections of the control diet and IBATi groups were free of lipid droplets, the HFD group exhibited increased accumulation of lipid droplets, indicating hepatic steatosis. Conversely, this increase was suppressed in the HFD plus IBATi group (Fig. S1C and D). The hepatic crown-like structure, which is one of the unique histological features of NAFLD, was only found in liver sections of the HFD group (Fig. S1E). The NAFLD activity score (NAS) increased significantly from 0.33 ± 0.58 (mean ± SD) in the control diet group to 7.67 ± 0.58 in the HFD group; this score was changed to 1.67 ± 1.55 in the HFD plus IBATi group at 12 weeks of treatment (Fig. S1F). However, histopathological fibrosis was not observed in liver sections of any of the treatment groups at the end of the experiments (Fig. S1G). These results indicated that IBATi prevented body and liver weight gain and improved hepatic steatosis in HFD mice.

10.1128/mBio.01155-21.1 FIG S1 Effects of IBATi on HFD-induced NAFLD. (A) Time course of mouse body weights and liver weight/body weight ratios of the four treatment groups (*n* = 6). (B) Representative photographs of mice and livers from each treatment group after 12 weeks. (C) Representative hematoxylin and eosin (H&E)-stained liver sections of each treatment group. (D) Representative oil red O-stained liver sections of each treatment group. (E) The hepatic crown structure in the liver detected only in liver sections from the HFD group. (F) NAFLD activity scores (NAS) of liver sections of each treatment group. (G) Fibrosis stage of each treatment group. Significant differences compared with the control diet group are denoted with an asterisk (*P < *0.05), and those with the HFD group are denoted with a dagger symbol (*P < *0.05). Download FIG S1, TIF file, 2.8 MB. Copyright © 2021 Matsui et al. 2021 Matsui et al. https://creativecommons.org/licenses/by/4.0/ This content is distributed under the terms of the Creative Commons Attribution 4.0 International license.

### Biochemical analysis of serum and mRNA expression in the liver and terminal ileum of all treatment groups.

Serum samples obtained from each treatment group after 12 weeks were assayed using enzyme-linked immunosorbent assay (ELISA). Serum aspartate aminotransferase (AST) and alanine aminotransferase (ALT) concentrations were higher in the HFD group than in the control diet and IBATi groups. When HFD mice were treated with IBATi, these increases were prevented. Biochemical lipid measurements confirmed high levels of total cholesterol, low-density lipoproteins (LDL), high-density lipoproteins (HDL), and triglycerides in the sera of the HFD group compared to the control diet and IBATi groups. Serum levels of total cholesterol and LDL were significantly lower in the HFD plus IBATi group. Serum levels of total BAs in the HFD group did not differ from those in the HFD plus IBATi group (Fig. S2A).

10.1128/mBio.01155-21.2 FIG S2 Biochemical parameters from each treatment group. (A) Biochemical parameters of sera from each treatment group (*n* = 6) after 12 weeks assayed using ELISA. (B and C) Hepatic mRNA (B) and ileal mRNA (C) expression from each treatment group measured by RT-PCR. Significant differences compared with the control diet group are denoted with an asterisk (*P < *0.05), and those with the HFD group are denoted with a dagger symbol (*P < *0.05). (D) Fecal bile acid pool composition profiling of each group of mice was assayed by mass spectrometry. Download FIG S2, TIF file, 2.8 MB. Copyright © 2021 Matsui et al. 2021 Matsui et al. https://creativecommons.org/licenses/by/4.0/ This content is distributed under the terms of the Creative Commons Attribution 4.0 International license.

Previous studies reported reduced hepatic cholesterol 7-a-monooxygenase (*Cyp7a1*) and increased ileal *Fgf15* mRNA expression in NAFLD model mice (6, 15). Therefore, we analyzed hepatic and ileal mRNA expression in each treatment group. Although *Cyp7a1* expression in livers of the HFD group was decreased, treatment with IBATi increased its expression up to 3-fold in livers of the HFD group. Cholesterol 8-a-monooxygenase (*Cyp8a1*) and farnesoid X receptor (*Fxr*) mRNA expression in livers did not differ among treatment groups. In the terminal ileum, *Fgf15* mRNA expression was elevated in the HFD group, which was prevented with IBATi treatment (Fig. S2B and C). These results indicated that IBATi treatment improved hepatic steatosis and liver dysfunction. Hepatic *Cyp7a1* mRNA expression was suppressed, and ileal *Fgf15* mRNA expression was increased in the HFD group. However, treatment with IBATi ameliorated these mRNA expression changes in HFD mice. In Fig. S2D, amounts of the FXR agonists omega-muricholic acid (ωMCA) and deoxycholic acid (DCA) were high in the feces from HFD mice. Although the amount of DCA in feces from HFD plus IBATi mice was as same as from HFD mice, ωMCA was significantly reduced in feces from HFD plus IBATi mice. Additionally, β-muricholic acid (βMCA) and lithocholic acid (LCA) were also significantly reduced.

### Effects of IBATi on HFD-induced gut microbiota dysbiosis.

**FIG 1:**
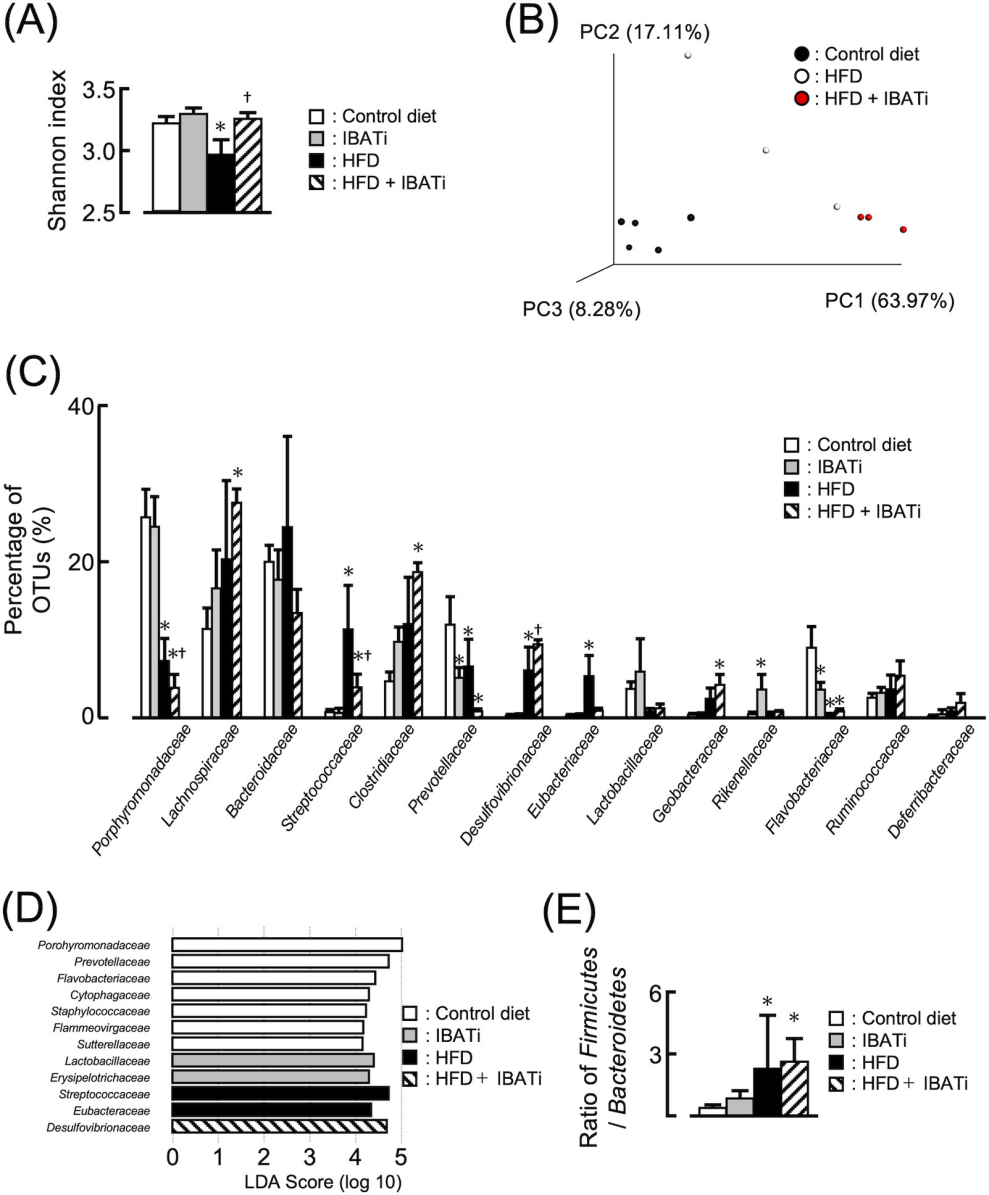
Effects of IBATi administration on gut microbiota. (A) Shannon index (OTU evenness estimation) in each treatment group. (B) PCoA plot of unweighted Bray-Curtis data for each treatment group. (C) Microbial composition at the family level in each group. (D) Relative abundance at the family level by linear discriminant analysis effect size (LEfSe) in each group. (E) Ratio of *Firmicutes* to *Bacteroidetes* in each group. Values are expressed as the mean ± SEM of three or five mice in each group. Significant differences compared with the control diet group are denoted by an asterisk (*P < *0.05), and those with the HFD group are denoted by a dagger symbol (*P < *0.05).

Given that the intestinal BA composition was likely to have altered the intestinal bacterial population, 16S rRNA sequencing was used to characterize the effect of IBATi on HFD-induced gut microbiota dysbiosis. In total, 166 operational taxonomic units (OTUs) were obtained from α-diversity among the control diet, HFD, IBATi, and HFD plus IBATi groups using different indices for the observed species and the Shannon index. The Shannon index was significantly lower in the HFD group than the control diet and IBATi groups, which was prevented by IBATi treatment (Fig. 1A).

Subsequently, the overall microbiota community structure of the control diet, HFD, and HFD plus IBATi groups were calculated using β-diversity indices for unweighted Bray-Curtis distances. Principal-coordinates analysis (PCoA) revealed significant differences in the microbiota structure of the control diet, HFD, and HFD plus IBATi groups (permutational multivariate analysis of variance [PERMANOVA], *P = *0.001) (Fig. 1B). HFD was associated with a higher proportion of *Streptococcaceae* (Fig. 1C). Relative abundances at the family level by linear discriminant analysis effect size (LEfSe) are shown in Fig. 1D. The ratio of *Firmicutes* to *Bacteroidetes* was increased in HFD groups compared to control diet groups (Fig. 1E). Additionally, relative abundances at the genus and species levels are shown in Fig. S3A and B. These results suggested that IBATi treatment changed the reduced gut microbiota diversity and microbiota dysbiosis induced by HFD.

10.1128/mBio.01155-21.3 FIG S3 Relative abundance at genus and species levels by LDA effect size (LEfSe) (A and B) Relative abundances from control diet, IBATi, HFD, and HFD plus IBATi groups at genus (A) and species (B) levels were analyzed by LEfSe. (C and D) Relative abundances from FMT mice from the HFD mice and FMT mice from the HFD plus IBAT mice at genus (C) and species (D) levels by LEfSe. (E and F) Relative abundances from control mice and IBAT-Tx mice at genus (E) and species (F) levels by LEfSe. Download FIG S3, TIF file, 2.8 MB. Copyright © 2021 Matsui et al. 2021 Matsui et al. https://creativecommons.org/licenses/by/4.0/ This content is distributed under the terms of the Creative Commons Attribution 4.0 International license.

From all FASTA files in metagenomic analysis of normal mice, HFD mice, IBATi mice, and HFD plus IBATi mice, we extracted sequences that were determined as *Streptococcaceae* at the family level. Each sequence had a similarity of 99% or more in the sequence of V4 region using the Greengenes database version 13.8. Three sequences were extracted, but almost one sequence (246 bp) was determined to be *Streptococcaceae* in this study (Data Set S1). The percentage of this sequence in all OTUs of each sample was significantly increased in HFD mice and decreased in HFD plus IBATi group of mice. The bacteria whose sequence (246 bp) was 100% query covered were examined using Basic Local Alignment Search Tool (BLAST) in National Center for Biotechnology Information (NCBI). In the results, the bacteria whose all of the total 246 bp matched were Lactococcus lactis (taxonomy ID 1358), Lactococcus lactis subsp. *cremoris* (taxonomy ID 1359), Lactococcus taiwanesis (taxonomy ID 1151742), and Lactococcus kimuchii (taxonomy ID 2568007). However, most of the bacteria were Lactococcus lactis and Lactococcus lactis subsp. *cremoris*. Next, we investigated the influence of these strains on bile acid metabolisms using Kyoto Encyclopedia of Genes and Genomes (KEGG) organisms (https://www.genome.jp/kegg-bin/show_brite?br08601.keg+lgr). There were total of 13 strains of Lactococcus lactis and Lactococcus lactis subsp. *cremoris* registered in KEGG, including subspecies, and none of them showed promotion of the synthesis of secondary bile acids in KEGG pathway analysis. On the other hand, it has been reported that Lactococcus lactis and Lactococcus lactis subsp. *cremoris* may have bile acid resistance (16), and those with bile acid resistance express bile salt hydrolase (BSH) (17). It is known that primary bile acids are metabolized to secondary bile acids by bacteria having bile acid 7α-dehydroxylase after being deconjugated by BSH. Lactococcus lactis and Lactococcus lactis subsp. *cremoris* do not directly synthase secondary bile acids but may be involved in the increase in secondary bile acids in feces. To support this, the percentages of bacteria with bile acid dehydroxylase increased in the feces of HFD mice in this study. The *Lachnospiraceae* was increased in feces of HFD mice, and the sequences which were determined as *Lachnospiraceae* were extracted from FASTA file. This sequence was matched to *Dorea* by the Greengene database v13.8, and *Dorea* is known to have produced secondary bile acids by dehydroxylation (18). That is, these Lactococcus lactis and Lactococcus lactis subsp. *cremoris* may eventually be involved in the increase of secondary bile acids. In the future, it will be necessary to research the influence of the combination of these bacteria.

10.1128/mBio.01155-21.4 DATA SET S1 From all FASTA files in metagenomic analysis, this sequence extracted was almost determined as *Streptococcaceae* at the family level. Download Data Set S1, DOC file, 0.02 MB. Copyright © 2021 Matsui et al. 2021 Matsui et al. https://creativecommons.org/licenses/by/4.0/ This content is distributed under the terms of the Creative Commons Attribution 4.0 International license.

### Effect of fecal microbiome transplantation on NAFLD mice.

**FIG 2:**
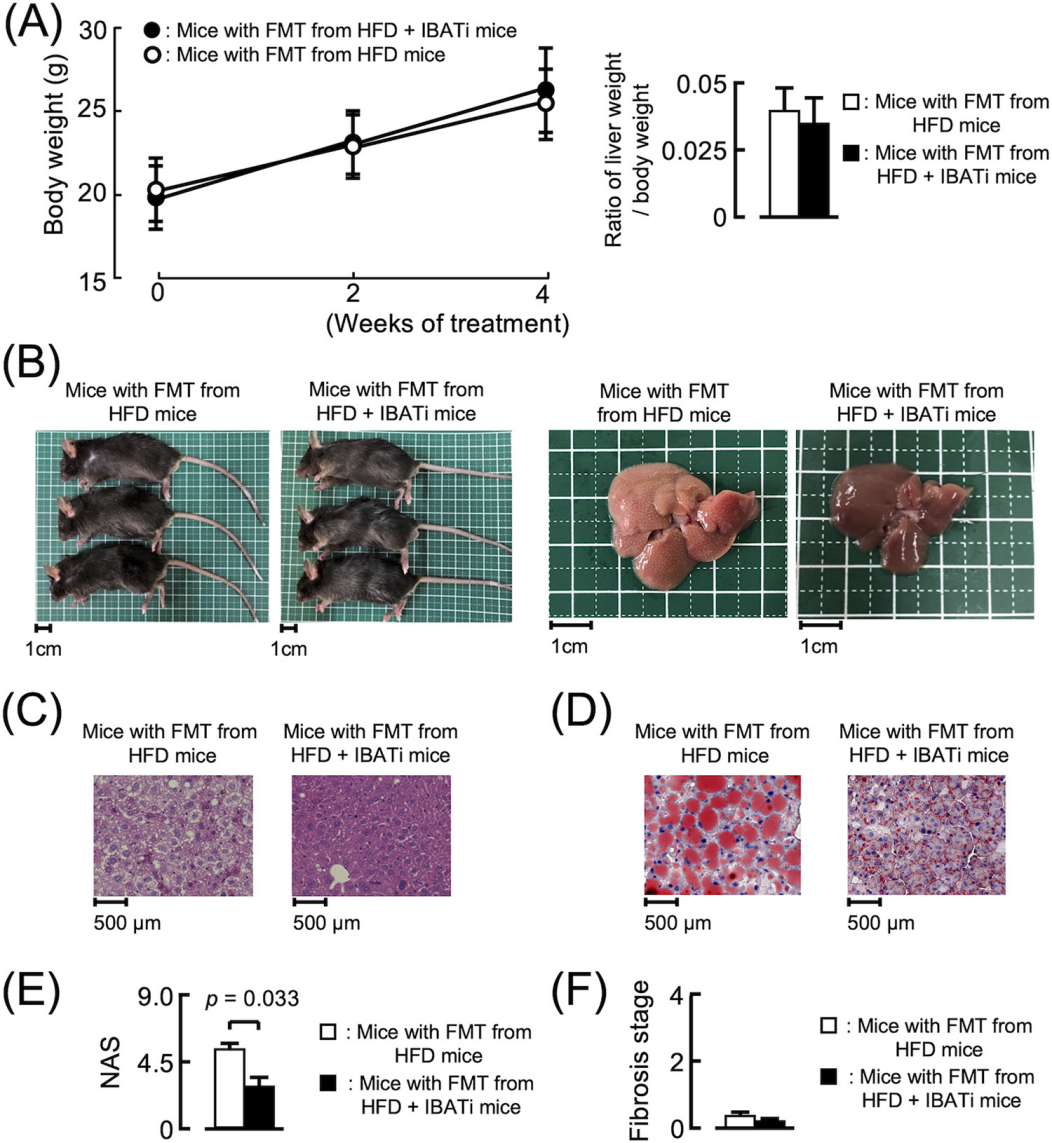
Effects of fecal microbiota transplantation (FMT) from HFD plus IBATi mice on hepatic steatosis. (A) Time course of mouse body weights and liver weight/body weight ratios of groups (*n* = 6) who received FMT from HFD mice or HFD plus IBATi mice. (B) Representative photographs of mice and livers from each group. (C) Representative hematoxylin & eosin (HE)-stained liver sections of each group. (D) Representative oil red O-stained liver sections of each group. (E) NAFLD activity scores (NAS) of liver sections of each group. (F) Fibrosis stage of each group.

**FIG 3:**
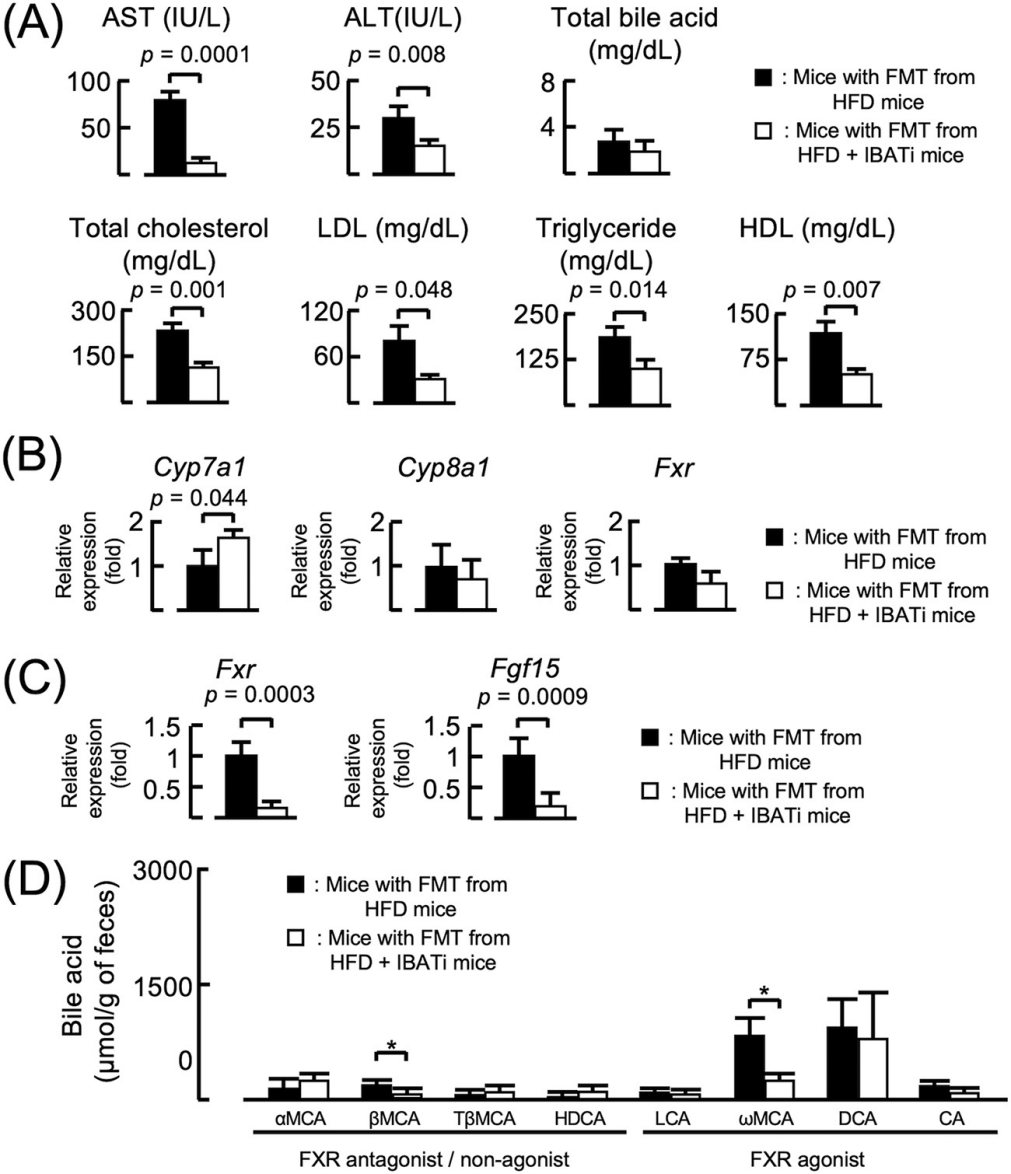
Biochemical parameters of mice with fecal microbiota transplantation (FMT). (A) Serum biochemical parameters from groups (*n* = 6) who received FMT from HFD mice or HFD plus IBATi mice assayed using ELISA. (B and C) Hepatic mRNA (B) and ileal mRNA (C) expression from each group measured by reverse transcription-quantitative PCR (RT-PCR). (D) Fecal bile acid pool composition profiling of each group of mice was assayed by mass spectrometry.

To establish the mechanism underlying improvement in NAFLD induced by IBATi, we recolonized antibiotic (ATB) solution-treated mice reared in specific-pathogen-free conditions by fecal microbiome transplantation (FMT) using stool from HFD or HFD plus IBATi groups. After FMT, both groups were fed HFD for 4 weeks. The body weights of both groups increased similarly, and the liver weight/body weight ratios of the groups did not differ at the end of the experiment (Fig. 2A). Fatty changes were observed in livers excised from mice with FMT from HFD mice, whereas this change was not detected in livers of mice with FMT from HFD plus IBATi mice (Fig. 2B). Lipid droplets were observed in histological liver sections of mice with FMT from HFD mice but not in mice with FMT from HFD plus IBATi mice (Fig. 2C and D). The average NAS of livers from mice with FMT from HFD mice (5.67 ± 2.08) was significantly higher than that from mice with FMT from HFD plus IBATi mice (2.67 ± 0.58) (*P = *0.033) (Fig. 2E). As shown in Fig. S1G, fibrosis was not observed in liver sections of either FMT group (Fig. 2F). AST, ALT, total cholesterol, triglycerides, LDL, and HDL levels in sera from mice with FMT from HFD plus IBATi mice were lower than those from mice with FMT from HFD mice (Fig. 3A). In addition, mice with FMT from HFD plus IBATi mice exhibited elevated hepatic *Cyp7a1* and reduced ileal *Fgf15* mRNA expression (Fig. 3B and C). In Fig. 3D, ωMCA in feces from mice with FMT from HFD plus IBATi mice was significantly reduced compared to the feces from mice with FMT from HFD mice.

**FIG 4:**
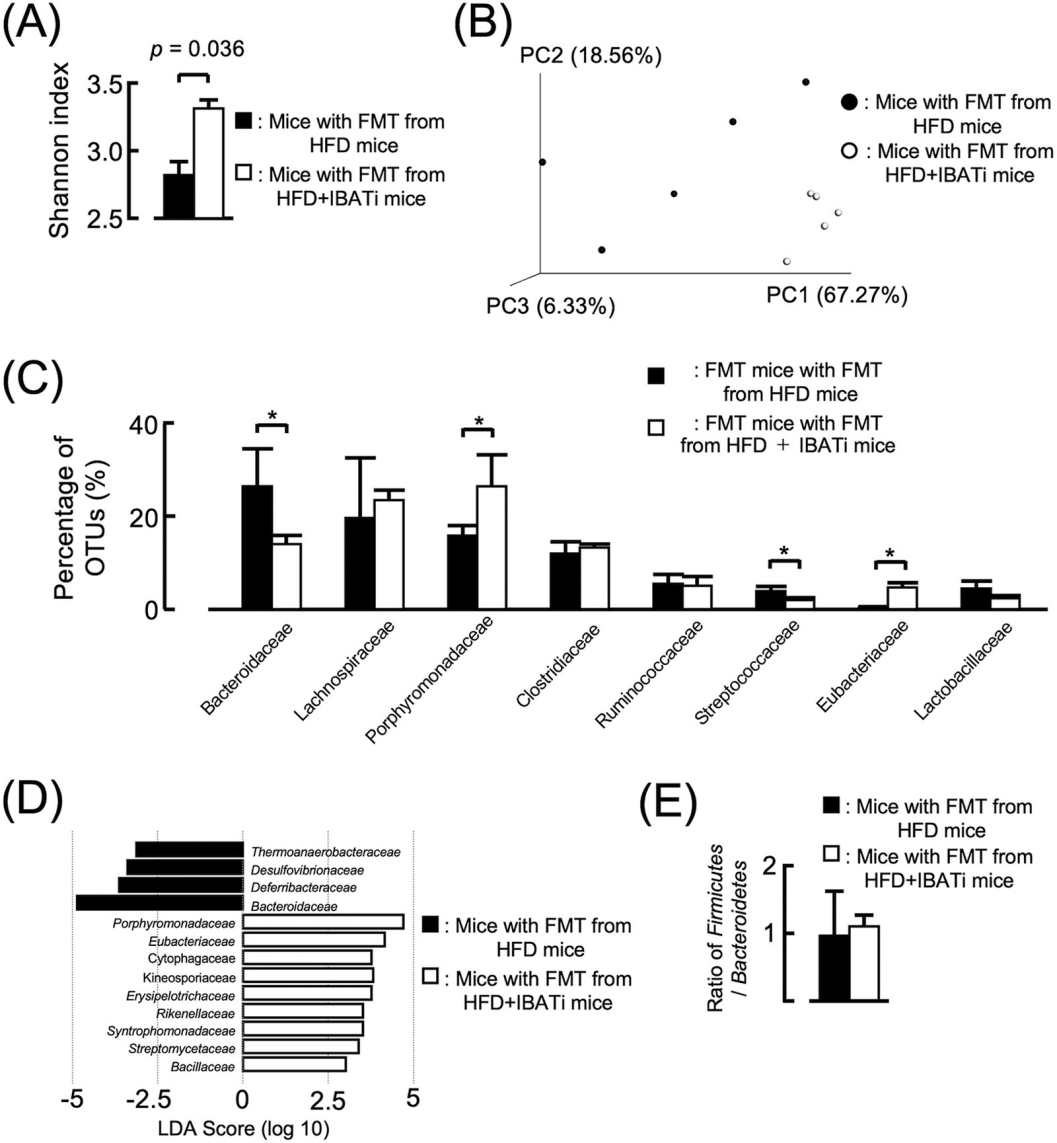
Influence of fecal microbiota transplantation (FMT) on gut microbiota. (A) Shannon index (OTU evenness estimation) in groups who received FMT from HFD mice or HFD plus IBATi mice. (B) PCoA plot of unweighted Bray-Curtis data for both FMT groups. (C) Microbial composition at the family level in each FMT group. (D) Relative abundance at the family level by LEfSe in each FMT group. (E) Ratio of *Firmicutes*/*Bacteroidetes* in each FMT group. Values are expressed as the mean ± SEM of three mice in each group.

**FIG 5:**
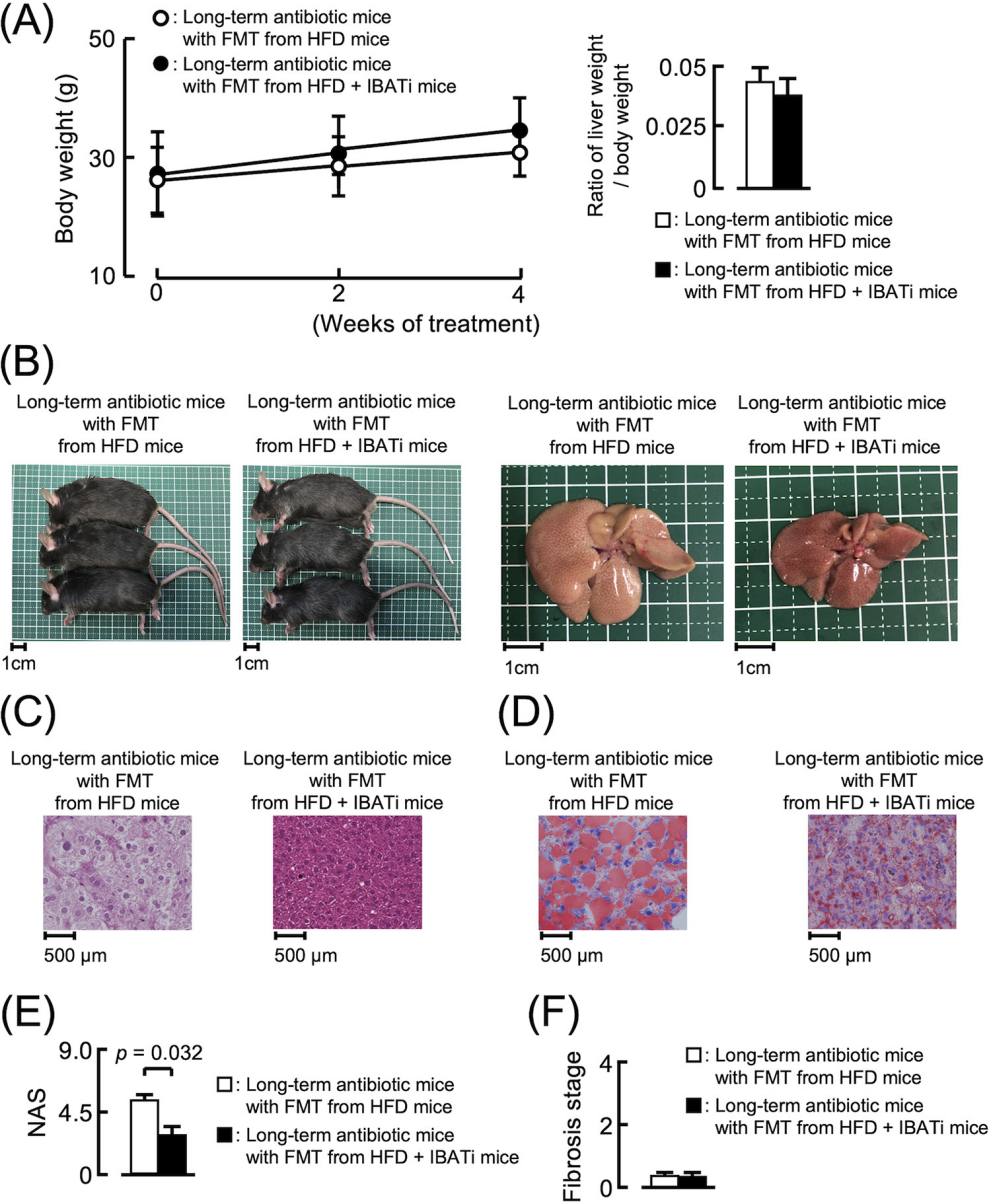
Effects of fecal microbiota transplantation (FMT) from HFD plus IBATi mice on hepatic steatosis in long-term antibiotics mice. Mice receiving long-term antibiotics (6 weeks) were transplanted with gut microbiota from HFD or HFD plus IBATi groups of mice (*n* = 5 each), and both groups of mice were fed HFD for 4 weeks. (A) Time course of mouse body weights and liver weight/body weight ratios (*n* = 5). (B) Representative photographs of mice and livers from both groups after 4 weeks. (C) Representative hematoxylin and eosin (H&E)-stained liver sections of both groups. (D) Representative oil red O-stained liver sections of both groups. (E) NAFLD activity scores (NAS) of liver sections of both groups. (F) Fibrosis stage of both groups.

The α-diversity in mice with FMT from HFD mice was reduced to the same level as that in HFD mice, but the α-diversity in mice with FMT from HFD plus IBATi mice was maintained even when fed HFD (Fig. 4A). β-Diversity differed significantly between the two groups (PERMANOVA, *P = *0.007) (Fig. 4B). The relative abundance of *Bacteroidaceae* at the family level was decreased in mice with FMT from HFD plus IBATi mice (*P = *0.00069). The percentage of *Streptococcaceae* in total OTUs from FMT mice with FMT from HFD plus IBATi was decreased from that of FMT mice with FMT from HFD mice (Fig. 4C). However, based on LEfSe, *Streptococcaceae* was not associated with mice with FMT from HFD mice (Fig. 4D). The ratio of *Firmicutes* to *Bacteroidetes* was the same in both groups (Fig. 4E). We additionally examined the effect of elobixibat in mice receiving long-term antibiotics. Mice receiving long-term antibiotics (6 weeks) were transplanted with gut microbiota from HFD or HFD plus IBATi groups of mice, and both groups of mice were fed HFD for 4 weeks. The gut microbiota fecal transplant from HFD plus IBATi mice prevented hepatic steatosis, even if mice receiving long-term antibiotics were fed HFD (Fig. 5). These results indicated that the gut microbiota fecally transplanted from HFD plus IBATi mice prevented hepatic steatosis caused by HFD.

### Therapeutic effect of IBATi against NAFLD in HFD mice.

**FIG 6:**
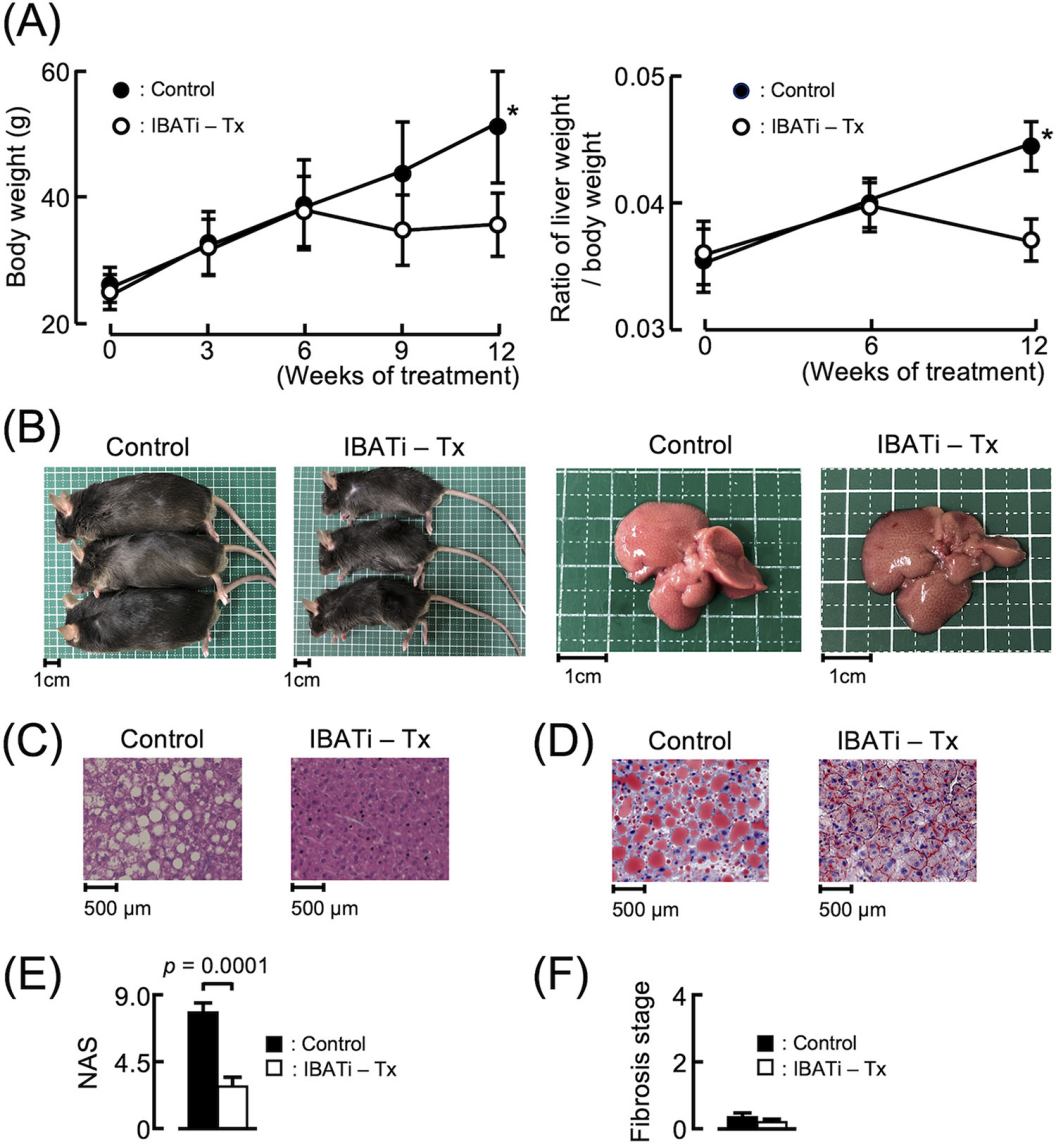
Therapeutic effects of IBATi on HFD-induced NAFLD. (A) Time course of mouse body weights and liver weight/body weight ratios of HFD mice treated without (control) and with IBATi (IBATi-Tx) (*n* = 6). (B) Representative photographs of mice and livers from each group. (C) Representative hematoxylin & eosin (HE)-stained liver sections from each group. (D) Representative oil red O-stained liver sections from each group. (E) NAFLD activity score (NAS) of liver sections from each group. (F) Fibrosis stage of each group.

**FIG 7:**
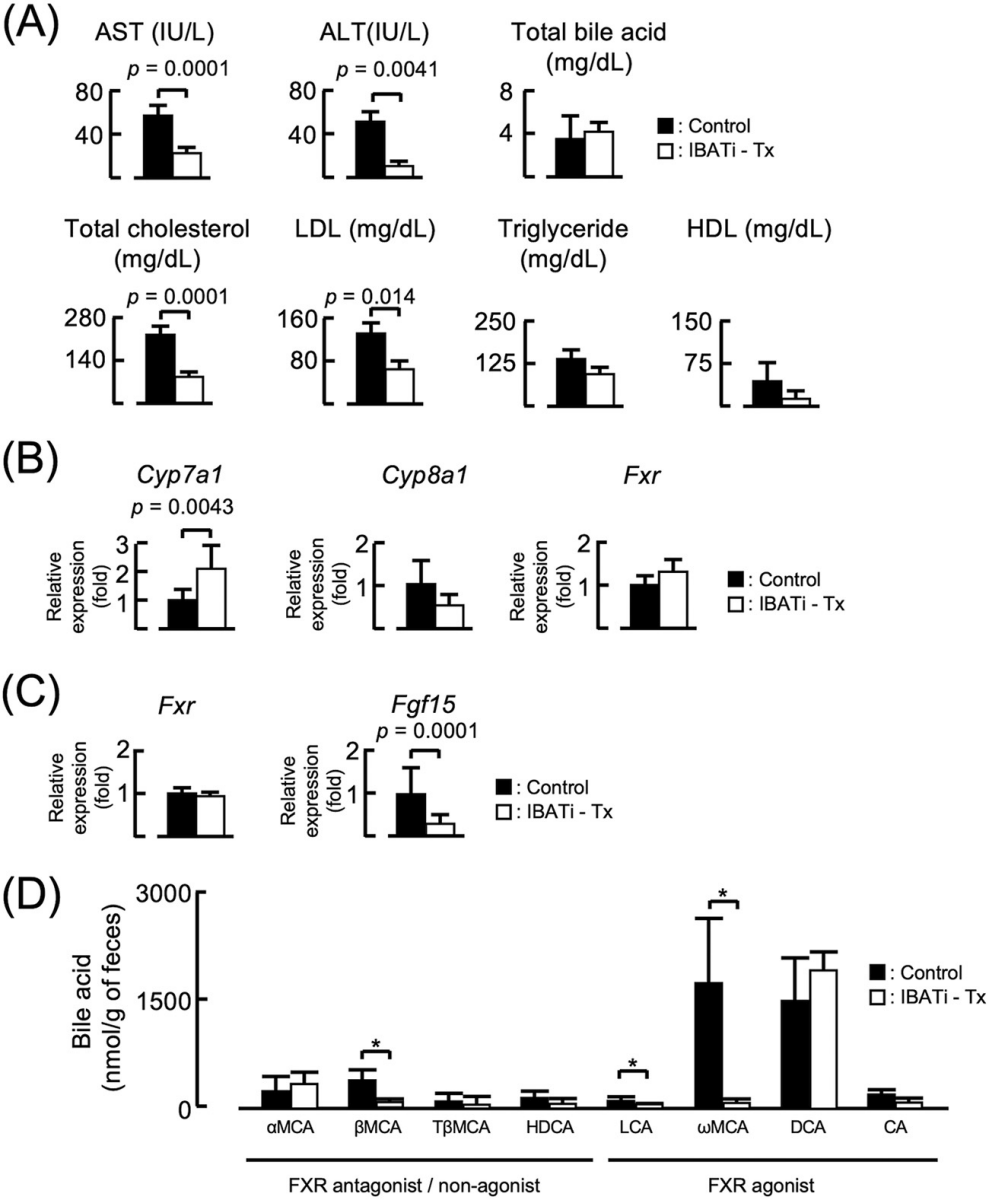
Biochemical parameters of IBATi-TX mice. (A) Biochemical parameters of sera from HFD mice treated without (control) and with IBATi (IBATi-Tx) assayed using ELISA (*n* = 6). (B and C) Hepatic mRNA (B) and ileal mRNA (C) expression in each group measured by RT-PCR. (D) Fecal bile acid pool composition profiling of each group of mice was assayed by mass spectrometry.

Next, we tested the therapeutic effect of IBATi in HFD mice. Six-week-old mice were fed HFD for 12 weeks and received IBATi treatment (IBATi-Tx group) between weeks 6 to 12. The control group was treated with the same dose of phosphate-buffered saline (PBS). The IBATi-Tx group did not exhibit the same increase in body weight as the control group; their weights remained stable with IBATi treatment. Liver weight/body weight ratios in the control group increased, but those in the IBATi-Tx group decreased after IBATi treatment (Fig. 6A). Fatty changes observed in the macroscopic photographs of liver sections from the control group were prevented in the IBATi-Tx group (Fig. 6B to D). The average NAS of livers from the IBATi-Tx group (1.66 ± 0.57) was improved compared with the control group, and fibrosis was not detected in either group (Fig. 6E and F). Lower serum AST, ALT, total cholesterol, and LDL levels were detected in the IBATi-Tx group. Other biological parameters in the sera did not differ between treatment groups (Fig. 7A). Elevated hepatic *Cyp7a1* and reduced ileal *Fgf15* mRNA expression were detected in mice after IBATi treatment (Fig. 7B and C). In Fig. 7D, ωMCA in feces from the IBATi-Tx group of mice was significantly reduced.

**FIG 8:**
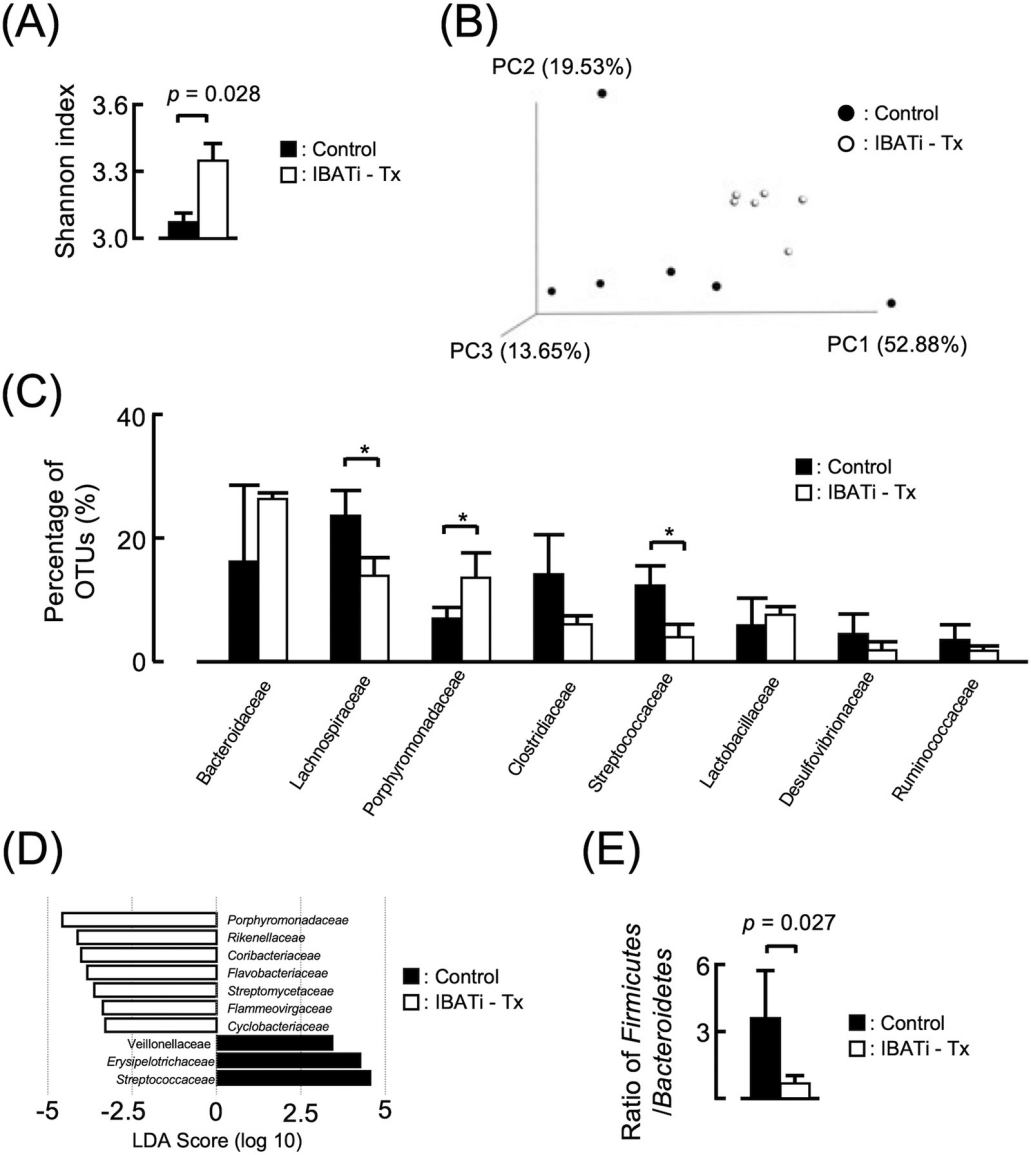
Effects of IBATi treatment on gut microbiota. (A) Shannon index (OTU evenness estimation) of HFD mice treated without (control) and with IBATi (IBATi-Tx). (B) PCoA plot of unweighted Bray-Curtis data for both groups. (C) Microbial composition at the family level in both groups. (D) Relative abundance at family level by LEfSe in both groups. (E) Ratio of *Firmicutes*/*Bacteroidetes* in both groups. Values are expressed as the mean ± SEM of three mice in each group.

Comparing the gut microbiota in both treatment groups, the α-diversity in the IBATi-Tx group was changed to the same levels as the control diet group, and β-diversity differed significantly between treatment groups (PERMANOVA, *P = *0.036) (Fig. 8A and B). The relative abundance at the family level of *Streptococcaceae* was increased in the IBATi-Tx group (Fig. 8C and D). Moreover, the ratio of *Firmicutes* to *Bacteroidetes* was significantly decreased after IBATi-Tx treatment (Fig. 8E). Thus, the therapeutic effect of IBATi against hepatic steatosis was reproduced in HFD-fed mice.

## DISCUSSION

Weight reduction can lead to significant improvements in the histological activity of NAFLD (19). Two mechanisms are associated with improvement of NAFLD, weight reduction and changing gut microbiota dysbiosis (20, 21). Weight reduction was not detected in mice with FMT from HFD plus IBATi mice in the current study, but NAFLD activity scores improved in the same group of mice. From these results, the improvement induced by IBATi in NAFLD model mice was hypothesized to be the result of changing gut microbiota dysbiosis, not weight reduction.

BAs are synthesized in hepatocytes via cytochrome P450 (CYP)-mediated oxidation of cholesterol to primary BAs through “classical” and “alternative” pathways in which CYP7A1 is the major enzyme, and BAs are secreted into the small intestine, where they facilitate the absorption of dietary lipids. The majority of BAs are reabsorbed from the intestine, returned to the liver via the portal venous system, and resecreted into the bile. The enterohepatic circulation of BAs is an extremely efficient process; less than 10% of intestinal BAs escape reabsorption and are eliminated in feces (22). IBAT is essential for efficient intestinal absorption of BAs, and mechanisms of alternative BA absorption are unable to compensate for the loss of IBAT function (23). In ileum enterocytes, activation of farnesoid X receptor (FXR) by BAs reabsorbed by IBAT releases FGF15 (the mouse ortholog of human FGF19) into portal circulation. FGF15 binds its receptor fibroblast growth factor receptor 4 (FGFR4) and inhibits CYP7A1, thus repressing BA synthesis in hepatocytes (7). Here, we identified IBATi as an important regulator of hepatic lipid metabolism through the gut microbiota in addition to its role in BA metabolism. It has been reported that the gut microbiota directly inhibits IBAT-dependent intestinal bile acid reabsorption (24). In particular, enteropathogenic Escherichia coli (EPEC) directly inhibits IBAT, which is dependent on intact bacterial effector molecules into host cells via the type 3 secretion system (25). However, EPEC was not detected in the feces of HFD mice in this study. The mechanism by which the gut microbiota regulates IBAT remains largely unknown and there may be other bacteria with similar capabilities. This area of investigation, which is in its infancy, may merit further research.

In the ileum, IBAT mediates most of the BA uptake from the gut lumen across the apical brush border membranes of enterocytes, whereas the organic solute transporter αβ (OST α/β) plays a central role in mediating intestinal basolateral BA export. In order to inhibit BA reabsorption, either IBAT or OST may be controlled. Both Asbt-null and Ostα-null mice reportedly exhibited impaired ileal BA absorption, decreased return of BAs to the liver, and decreased BA pool size (26, 27). However, hepatic *Cyp7a1* mRNA expression and BA synthesis were paradoxically suppressed in Ostα-null mice, secondary to elevated ileal FGF15 production (28). Blocking intestinal absorption of BAs at the apical brush border membranes of enterocytes by IBATi suppressed ileal *Fgf15* mRNA expression, induced hepatic BA synthesis, and reduced plasma cholesterol levels. However, the broad substrate specificity of OST α/β raised the concern that dietary constituents, dietary supplements, or drugs could act as OST α/β inhibitors to slow BA export, activate FXR to induce intestinal FGF15 expression, and raise plasma cholesterol levels (23). Because these reports were significant with regard to the mechanism of BA sequestrant activity, we investigated the effect of IBATi against NAFLD.

Alterations in the gut microbiota were associated with HFD in this study, but little is known about the host factors that induced this alteration in the microbial population. HFD-induced BA secretion was first proposed in 2011 by Islam et al. (29) as a driving force in shaping obesity-associated gut microbial composition. Cholic acid (CA) is the most abundant BA in human biliary secretions, which is enhanced by HFD to facilitate lipid digestion. The most important secondary BA is deoxycholic acid (DCA), which arises from CA. The conversion to secondary BAs is mediated by a 7α-dehydroxylation reaction catalyzed by some species of *Clostridium*; nearly 100% of CA is converted into DCA in the large intestine. An important feature of BAs is their strong antimicrobial activity (30). Physiological concentrations of CA and DCA in the intestine disturb the integrity of membranes as a result of their detergent effect, leading to the leakage of ions and cellular components and eventually cell death. DCA is one of the most potent antimicrobial BAs, with 10 times the bactericidal activity of CA. Thus, BAs seem to exert a strong selective pressure on the gut microbiota, and only microbial populations able to tolerate physiological concentrations of BAs can survive in the gut. This seems especially evident when administering HFD, causing increased intestinal flow of BAs (29). In this study, ωMCA was detected in feces from the HFD group of mice. ωMCA, which is synthesized by gut microbiota, activated FXR of ileum enterocytes. FGF15 produced from ileum enterocytes suppresses Cyp7a1 in the liver, and bile acid synthesis from cholesterol is inhibited. It is suggested that IBATi not only inhibits the reabsorption of bile acid but also changes the gut microbiota and suppresses the synthesis of ωMCA to improve the fatty liver.

There is growing concern that environmental factors, especially HFD, have altered the genetic composition and metabolic activity of the human microbiota (31), predominantly through changes in the relative abundance of two dominant bacterial divisions, *Firmicutes* and *Bacteroidetes*. At the family level, dramatic overgrowth of *Streptococcaceae* and *Enterobacteriaceae* has been observed in obese subjects (32). In this study, *Streptococcaceae* abundance was also increased in the gut microbiota of HFD mice, which decreased to the same level as that in control diet mice after FMT from HFD plus IBATi group mice. Due to the critical role of gut microbiota in NAFLD development, the promotion of microbiota composition changes to resemble a healthier profile may be desirable in clinical practice; focus on modulatory effects against dysbiosis and activation of the gut-liver axis is warranted. The administration of live bacteria in adequate amounts with potential health benefits for the host (i.e., probiotics) is a promising strategy to manage obesity-associated disorders. Bacteria belonging to *Lactobacillus*, Streptococcus, and *Bifidobacterium* genera are used most frequently as probiotics due to the growing body of literature supporting their health-promoting effects and their ability to integrate into the normal human gut microenvironment (33). In this study, IBATi promoted changes in the gut microbiota and impeded hepatic lipid accumulation. The protective effects of probiotics against NAFLD are not yet fully understood, although it has been proposed that production of antibacterial substances, enhancement of epithelial barrier function, and regulation of the immune system and subsequent intestinal inflammation may be implicated (9).

### Conclusions.

NAFLD is highly prevalent worldwide, but effective therapeutic agents for its treatment are lacking. HFD mice treated with IBATi exhibited significantly suppressed body weight gain, liver dysfunction, and serum LDL levels, and their NAFLD activity scores were significantly decreased. The reduced α-diversity in the gut microbiota induced by HFD was recovered by IBATi treatment. IBATi treatment also ameliorated the increased ileal *Fgf15* and decreased hepatic *Cyp7a1* mRNA levels in HFD mice. Fecally transported gut microbiota from HFD plus IBATi mice prevented hepatic steatosis caused by HFD, indicating that IBATi protected against hepatic steatosis by ameliorating gut microbiota dysbiosis in NAFLD model mice.

## MATERIALS AND METHODS

### Mice.

All animal experiments were carried out in compliance with the International Council for Laboratory Animal Science (ICLAS) Ethical Guideline for Researchers and Japanese regulations. The local institutional animal ethics board of Osaka Medical College approved all mouse experiments (approval number 2019-138). Five-week-old male C57BL/6 mice were obtained from Japan SLC, Inc. (Shizuoka, Japan). All animal experiments were performed at the animal facility in Osaka Medical College, where mice were housed under specific-pathogen-free conditions. After acclimation for 1 week, mice were divided into four groups (*n* = 6): two groups were fed the control diet (CE-2; CLEA Japan Inc., Tokyo, Japan), and two groups were fed HFD (HFD32; CLEA Japan, Inc.) for 12 weeks (34). For the IBATi and HFD plus IBATi groups, mice were treated with 2.5 μmol/kg of the oral IBATi elobixibat (EA Pharma Co. Ltd., Tokyo, Japan) in 0.2 ml PBS by daily oral gavage from 6 to 12 weeks (5). Mice in the control diet groups were subjected to the same oral dose of PBS. Body weight and food consumption were monitored weekly throughout the experiments.

### Fecal microbiota transplantation.

Elobixibat has only 2% bioavailability and is excreted from the body within 48 h after oral administration. When absorbed, elobixibat is protein bound in plasma (>99%), with a half-life of less than 4 h (35, 36). For fecal microbiota transplantation (FMT), feces were collected from the 12 week-HFD and HFD plus IBATi groups 48 h after the last oral administration of elobixibat and stored at −80°C. Six-week-old male C57/B6 mice (*n* = 10) were fed HFD for 3 weeks, after which they were treated with ATB solution containing ampicillin (1 mg/ml), streptomycin (5 mg/ml), and colistin (1 mg/ml) added to their sterile drinking water. Solutions and bottles were changed 3× per week. ATB treatment was discontinued 48 h before the first FMT treatment (37). FMT was performed by thawing fecal materials, and 500 μl of fecal suspension (50 mg/mouse) was then transferred by oral gavage into each ATB-treated recipient 3× every 2 days for 1 week. Mice were then fed HFD for an additional 4 weeks.

In some experiments, mice treated with ATB solution containing ampicillin (1 mg/ml), streptomycin (5 mg/ml), and colistin (1 mg/ml) added to their sterile drinking water for 6 weeks were transplanted with gut microbiota from HFD or HFD plus IBATi groups of mice (*n* = 5 each), and both groups of mice were fed HFD for 4 weeks.

### Metagenomic analysis of mouse feces.

Fresh feces were collected, placed into tubes, and stored at −80°C until further use. Bacterial genomic DNA was extracted from fecal samples as previously described (38), using a PureLink microbiome DNA purification kit (Thermo Fisher Scientific, Tokyo, Japan). Preparation of the library for DNA sequencing of the 16S RNA gene was conducted as previously described (39) using an Ion 16S Metagenomics kit (Thermo Fisher Scientific) to amplify hypervariable regions from the polybacterial samples according to the manufacturer’s protocol. Library preparation was performed using 50 ng pooled amplicons for each sample with the Ion Plus fragment library kit (Thermo Fisher Scientific). The StepOnePlus real-time PCR system (Thermo Fisher Scientific) was used to perform quantitative PCR amplification.

### Sequence data analysis.

Sequence data analysis was performed using QIIME v1.9.0 as previously described (39). Statistical differences (*P < *0.05) in the relative abundance of bacterial phyla and genera between groups were evaluated using Student’s paired *t* tests. Shannon phylogenetic diversity indices were calculated using the R phyloseq package and statistically analyzed using Kruskal-Wallis tests followed by Steel-Dwass *post hoc* tests. β-Diversity was estimated using Bray-Curtis distances between samples, visualized by PCoA, and statistically examined using permutational analysis of variance (PERMANOVA). The final figures were generated using QIIME v1.9.0. LEfSe was determined using default values (a value of 0.05 for both the factorial Kruskal-Wallis test among classes and the pairwise Wilcoxon test between subclasses; threshold value of 2.0 for the logarithmic LDA score for discriminative features).

### Histological analysis.

Mice were euthanized before tissue sampling, and liver was obtained from these mice. Liver tissue samples were fixed in 20% formalin and embedded in paraffin. Sections (4 μm thick) were cut and stained with H&E. Sections (8 μm thick) from frozen liver tissue samples were stained with oil red O. Histological features and the fat area in liver tissue sections were determined using a BZ-X710 fluorescence microscope (Keyence, Osaka, Japan). Blinded histological assessment of the slides was performed by an experienced hepatopathologist. Hepatic steatosis was evaluated by NAS (40), and hepatic fibrosis was evaluated by fibrosis stage (41).

### Biochemical analysis of serum and fecal bile acid pool composition profiling.

Blood was obtained from mice that were euthanized before tissue sampling, and serum was obtained from each mouse. Serum concentrations of AST, ALT, total BAs, total cholesterol, LDL, HDL, and triglycerides were measured using ELISA kits from Elabscience (Houston, TX, USA) and MyBioSource, Inc. (San Diego, CA, USA) following the manufacturers’ instructions. Fecal bile acid pool composition profiling of each group of mice was assayed by mass spectrometry (TechnoSuruga Laboratory Co., Ltd., Shizuoka, Japan).

### mRNA expression in the liver and terminal ileum.

Liver and terminal ileum samples were obtained from each group of mice after the anesthetization and prepared for analysis using an RNeasy minikit (Qiagen, Tokyo, Japan). The StepOnePlus real-time PCR system (Thermo Fisher Scientific) was used to perform quantitative PCR amplification. Hepatic mRNA expression (cholesterol 7-a-monooxygenase [*Cyp7a1*], cholesterol 8-a-monooxygenase [*Cyp8a1*], and farnesoid X receptor [*Fxr*]), and ileal mRNA expression (Fxr and fibroblast growth factor 15 [*Fgf15*]) were measured by real-time PCR using CYBR Green real-time PCR master mix (Thermo Fisher Scientific). The thermal cycling conditions included an initial denaturation step 95°C for 3 min, followed by 40 cycles at 95°C for 10 s and 56°C for 30 s.

### Statistical analysis.

All data are presented as the mean ± SEM. Body weight gain curves were analyzed using ANOVA, followed by Tukey’s multiple-comparison tests. Pearson’s rank correlation coefficient was used to analyze the correlation between gut microbiota. Independent samples *t* tests or Wilcoxon-Mann-Whitney U tests were used for differences between continuous variables. Statistical analyses were performed using Statistics Analysis Software (JMP Institute, Inc., Cary, NC, USA). All results were considered statistically significant at a *P* value of <0.05.

### Data availability.

The data sets used and/or analyzed in the study are available at NCBI BioProject (accession numbers PRJNA714695 and PRJNA714835).
