# Supplementary figures and images for: Ileal Bile Acid Transporter Inhibitor Improves Hepatic Steatosis by Ameliorating Gut Microbiota Dysbiosis in NAFLD Model Mice
Source: mBio. 2021 Jul 6;12(4):e01155-21. doi: 10.1128/mBio.01155-21 (PMC8406289; doi:10.1128/mBio.01155-21)

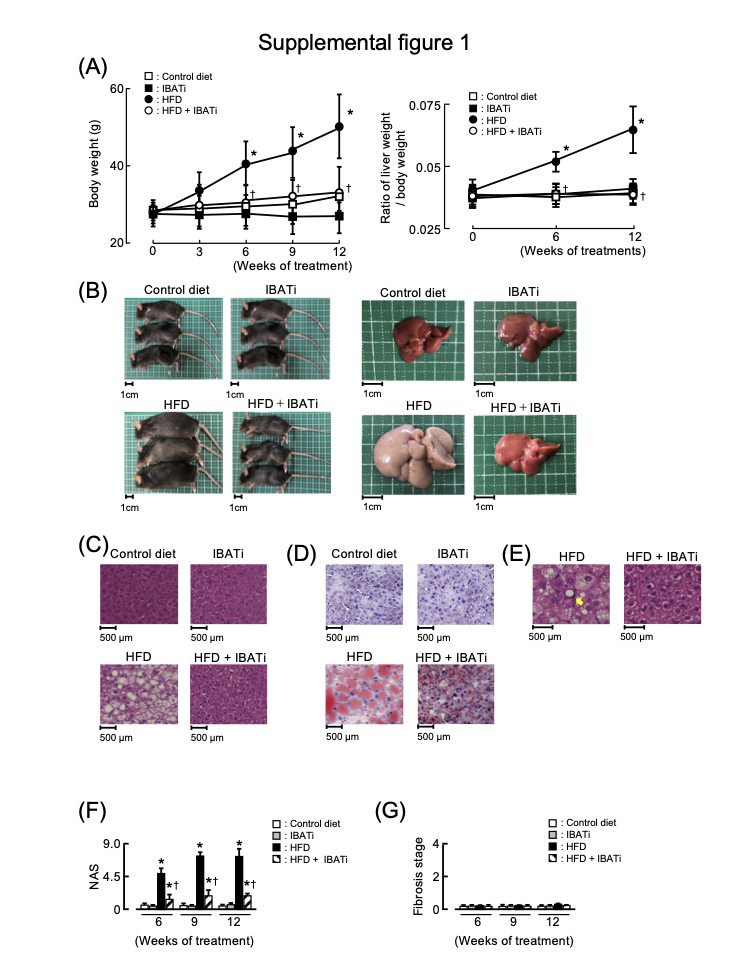

Supplement: FIG S1 [file mbio.01155-21-sf001.tif]

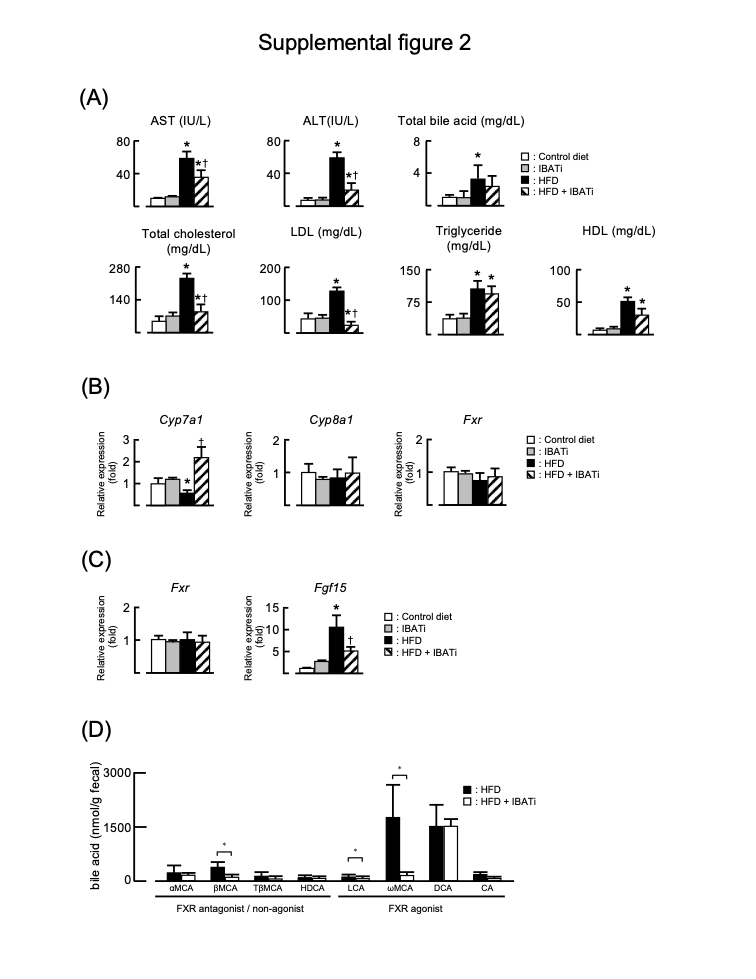

Supplement: FIG S2 [file mbio.01155-21-sf002.tif]

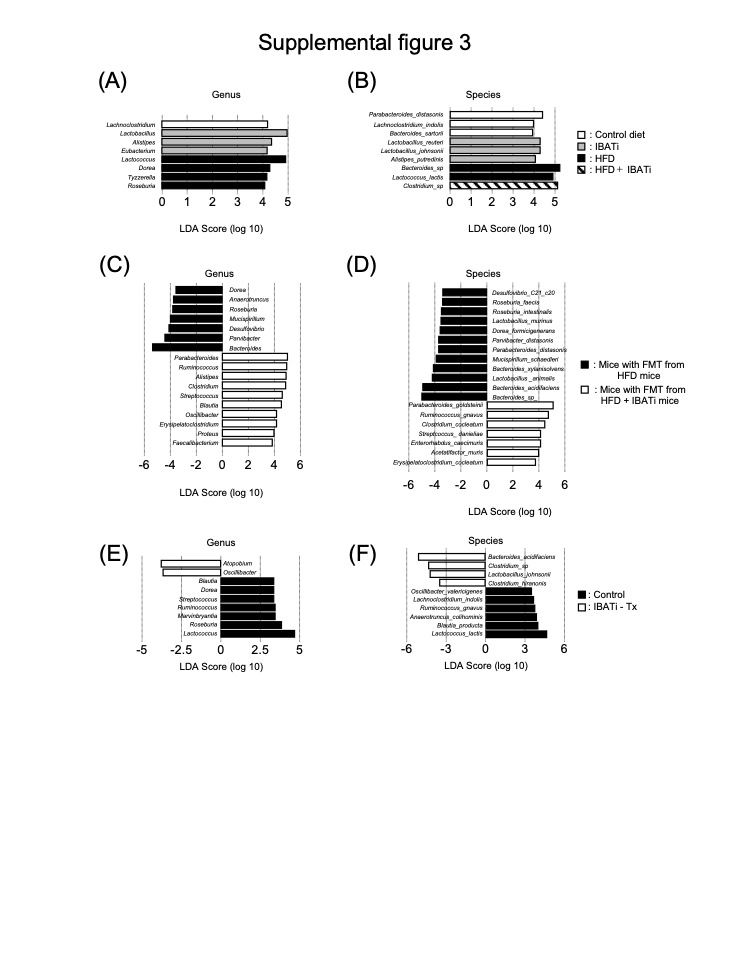

Supplement: FIG S3 [file mbio.01155-21-sf003.tif]
