# Supplementary material for: Ileal Bile Acid Transporter Inhibitor Improves Hepatic Steatosis by Ameliorating Gut Microbiota Dysbiosis in NAFLD Model Mice
Source: mBio. 2021 Jul 6;12(4):e01155-21. doi: 10.1128/mBio.01155-21 (PMC8406289; doi:10.1128/mBio.01155-21)
Supplement: DATA SET S1 [file mbio.01155-21-sd001.doc]

Supplemental Data 1

CGTAGGTCCCGAGCGTTGTCCGGATTTATTGGGCGTAAAGCGAGCGCAGGTGGTTTATTAAGTCTGGTGTAAAAGGCAGTGGCTCAACCATTGTATGCATTGGAAACTGGTAGACTTGAGTGCAGGAGAGGAGAGTGGAATTCCATGTGTAGCGGTGAAATGCGTAGATATATGGAGGAACACCGGTGGCGAAAGCGGCTCTCTGGCCTGTAACTGACACTGAGGCTCGAAAGCGTGGGGAGCAAA
